# An interaction network approach predicts protein cage architectures in bionanotechnology

**DOI:** 10.1073/pnas.2303580120

**Published:** 2023-12-07

**Authors:** Farzad Fatehi, Reidun Twarock

**Affiliations:** ^a^Departments of Mathematics, University of York, York YO10 5DD, United Kingdom; ^b^Department of Biology, University of York, York YO10 5DD, United Kingdom

**Keywords:** protein nanoparticle, interaction network, surface tiling, symmetry, AaLS pentamer cage

## Abstract

Protein nanoparticles play pivotal roles in many areas of bionanotechnology, including drug delivery, vaccination, and diagnostics. These technologies require control over the distinct particle morphologies that protein nanocontainers can adopt during self-assembly from their constituent protein components. The geometric construction principle of virus-derived protein cages is by now fairly well understood by analogy to viral protein shells in terms of Caspar and Klug’s quasi-equivalence principle. However, many artificial, or genetically modified, protein containers exhibit varying degrees of quasi-equivalence in the interactions between identical protein subunits. They can also contain a subset of protein subunits that do not participate in interactions with other assembly units, called capsomers, leading to gaps in the particle surface. We introduce a method that exploits information on the local interactions between the capsomers to infer the geometric construction principle of these nanoparticle architectures. The predictive power of this approach is demonstrated here for a prominent system in nanotechnology, the AaLS pentamer. Our method not only rationalises hitherto discovered cage structures but also predicts geometrically viable options that have not yet been observed. The classification of nanoparticle architecture based on the geometric properties of the interaction network closes a gap in our current understanding of protein container structure and can be widely applied in protein nanotechnology, paving the way to programmable control over particle polymorphism.

## Significance

Protein containers play important roles in nanotechnology applications, including diagnostics, drug delivery, and vaccination. These applications require programmable control over container size and stoichiometry. The seminal Caspar–Klug approach for the classification of virus architecture predicts nanocontainer geometry if protein subunits exhibit similar interaction types across the capsid surface. However, in many nanoparticles, there are identical protein subunits that do not interact with other subunits in the same way, thus presenting gaps in the container surface. We demonstrate that in these cases, the interaction network between the assembly units can be used to gain predictive control over the spectrum of particle morphologies. This paves the way to programmable control over particle polymorphism and informs the design of nanoparticles for specific applications.

Protein containers are ubiquitous in nature. Prominent examples are the viral protein shells, called capsids, that provide protection and transport for viral genomes between rounds of infection. Protein cages also serve vital functions in bacteria as microcompartments, or in prokaryotic cells, where encapsulins, ferritin, and lumazine synthase cages facilitate catalysis (1), intracellular trafficking (2), and transport (3, 4). Nanoparticles, either derived from these naturally occurring protein containers or de novo designed, play pivotal roles in a host of applications, including vaccine development (5, 6), cargo storage, drug delivery, gene therapy, and diagnostics (7, 8).

Viruses have evolved mechanisms to assemble specific geometric designs with high fidelity and efficiency. The vast majority of virus architectures exhibit icosahedral symmetry as a consequence of the principle of genetic economy (9), as this symmetry type allows container volume to be maximised without increasing the coding cost for its components. The additional volume in the confines of the capsid provides room to package genes supporting other functions, thus allowing viruses to gain more complexity with time. Insights into the geometric and mechanical properties of viral capsids afford a better understanding of viral life cycles. For example, buckling transitions from an initial spherical procapsid to the final icosahedral faceted shell have been shown to enhance a capsid’s tolerance of internal pressures (10), and models of thermal dissociation have elucidated the processes of viral assembly and disassembly (11). The structures of artificial nanoparticles, on the other hand, are not as well understood to date and exhibit a much wider spectrum of morphologies (6, 12–18). This is because they exhibit varying degrees of quasi-equivalence (19–22). There are cages in which a subset of the constituent protein subunits of the assembly unit of the capsid (capsomer) do not interact with neighbouring capsomers at all, resulting in larger gaps in the particle surface. As such gaps are biologically important, among others for diffusion-limited encapsulation of complementarily charged guest molecules (23), a better understanding of the geometric construction principle of such artificial protein cages is required. This is also an important step toward control over nanoparticle size and stoichiometry, enabling their manufacturing to be optimised, and their biophysical properties to be tuned for specific applications (12).

**Fig. 1.**
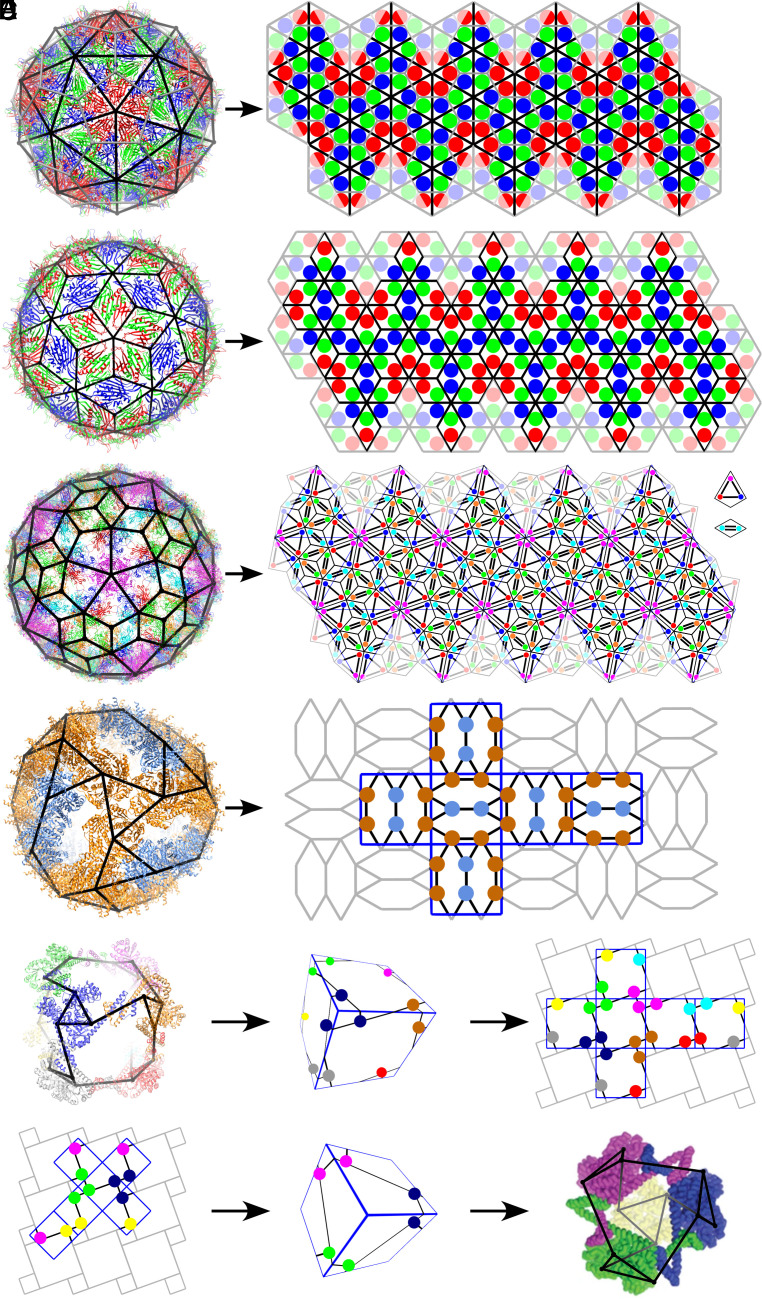
Tiling models of virus architecture. (*A*) A triangular tiling according to Caspar–Klug theory is a surface lattice model for the Pariacoto virus capsid (PDB: 1F8V); its dual, a hexagonal lattice (gray) also correctly models the relative positions of the capsid proteins. (*B*) A different surface lattice model, a rhomb tiling, is required to capture the relative CP positions in bacteriophage MS2 (PDB: 2MS2); rhombs are one-to-one with the protein dimers from which the capsid assembles. (*C*) The surface lattice of human papillomavirus (PDB: 3J6R) is made of two tiles, a kite and a rhomb, that represent trimer and dimer interactions in the capsid surface. Note that each CP interacts with CPs in other pentamers. (*D*) The pentamers in AaLS-neg, a protein cage made from 36 pentamers (PDB: 5MQ3), cannot be mapped onto pentagons in a surface lattice in which every tile has an interpretation in terms of protein positions. As a subset of CPs do not interact with proteins in other pentamers, this surface architecture exhibits gaps. However, the interaction network between capsomers (not protein subunits) can be represented as a tiling; its geometric information is exploited here to construct and classify alternative capsid architectures. (*E*) The interaction network of the protein cage reported in ref. 24 (PDB: 4QCC) can be mapped onto a cube; coloured vertices indicate the centres of mass of the CPs (*Middle*). This cubic surface can be embedded into a gyrated square tiling (*Right*). (*F*) Other embeddings of the cubic surface into the gyrated square tiling (*Left*) predict the morphologies of other cages that can assemble from the same protein units, such as the smaller cage reported in refs. 14 and 24 (*Right*).

The seminal Caspar–Klug (CK) theory was the first to propose quasi-equivalence as a geometric design principle for the structural organisation of icosahedral viruses (19). CK theory indicates capsid protein (CP) positions relative to surface triangulations, ascribing CPs to the polyhedral angles (corners) of the triangular tiles (Fig. 1*A*). The dual tilings, polyhedra with hexagonal and pentagonal faces akin to Buckminster Fuller’s domes (25), predict the same capsid layout, again assuming CPs to be located in the polyhedral angles of the faces (Fig. 1*A*, gray). Therefore, viral capsids are interchangeably modelled via hexagonal surface lattices and triangulations in CK theory. These geometric models predict protein positions correctly for capsids formed from pentagonal, hexagonal, or triangular capsomers, i.e., from pentamers, hexamers, and trimers. However, they do not accurately reflect the layout of capsids assembled from dimers (*SI Appendix*, Fig. S1). In the considered and some other cases, the tiles in the surface lattice are in a one-to-one correspondence with the biological units. We therefore represent bacteriophage MS2, which assembles from protein dimers, by a rhomb tiling (Fig. 1*B*), thus capturing the correct relative CP orientations. More general types of tilings are required for other capsomer types if viral capsids are formed from more than one type of protein unit (26).

A further complication arises for human papillomavirus (HPV), a capsid formed from 72 identical pentamers. Due to the crystallographic restriction (27) there is no all-pentamer surface lattice with more than 12 identical pentagonal tiles, implying that pentamers cannot be modelled by pentagonal tiles only. To rationalize this shell, a spherical tiling consisting of 72 pentamers and 60 narrow and 150 wide rhombs has been proposed where its vertices correspond to positions of protein centres (28). Recognising the need for tiles to have an interpretation in terms of biological units, we introduce two types of tiles for HPV, a rhomb and a kite, that are in a one-to-one correspondence with the two types of interactions mediated by the C-terminal arms: kites corresponding to three proteins forming a trimer interaction, and rhombs representing two proteins forming a dimer interaction (29) (Fig. 1*C*). This tiling is reminiscent of the Penrose tiling (30), an aperiodic tiling given in terms of kites and rhombs, and 3D Penrose tilings can also be used to approximate virus structure (31). There are other approaches modelling virus architecture, using a local rules approach (32) or a dodecahedral nets approach that associates the centres of mass of protein molecules in icosahedrally symmetric viral cages with the nodes of a chiral pentagonal tiling (33). However, the lack of a direct correspondence between tiles and biological units limits the predictive power of these approaches. They describe the layouts of different viruses built according to the same mathematical principle but do not allow for the classification of particle morphologies that can be formed from the same types of building blocks. By contrast, this is possible in viral tiling theory (VTT) as tiles have an interpretation in terms of assembly units or specific types of protein–protein interactions.

Protein nanoparticles exhibit a wider spectrum of morphologies than viruses. For example, in some nanoparticles, some of the protein subunits in a capsomer do not interact with other capsomers, as is the case for the 36-pentamer particles formed from *Aquifex aeolicus* lumazine synthase (AaLS) (23) (Fig. 1*D*).

In this case, neither capsomers nor interactions between individual CPs can be represented by tiles in a meaningful way. Of course, the particle surface can always be represented as a tessellation in terms of multiple copies of the fundamental domain (also called asymmetric unit) of the underlying symmetry group, in this case consisting of three Voronoi cells (*SI Appendix*, Fig. S2). However, in such mathematical representations, there is no clear biological interpretation of the lattice unit in terms of individual capsomers or interactions between protein subunits. Therefore, such an approach does not allow prediction of other possible cages made from AaLS pentamers.

In order to achieve this, we construct the interaction network between capsomers (rather than between their constituent protein subunits) (34). For this, the centres of mass (CoMs) of the capsomers are computed based on the coordinates in the PDB file. These then form nodes in a network in which connecting edges indicate interactions between capsomers (Fig. 1*D*). This coarse-grained topological descriptor of capsid architecture ignores the geometry of individual capsomers, and interactions formed by individual protein subunits, and is therefore distinct from the surface lattice models in VTT. However, the geometric structure of this interaction network can be embedded into a tiling. For this, the symmetry axes of the particle are aligned with those of a reference cube (*SI Appendix*, Fig. S3), and then, the cubic surface is embedded into a planar tiling that continues the interaction network periodically in the plane. This tiling can then be used to construct models for other particle types via different embeddings of the cubic surface, akin to the embedding of icosahedral surfaces into hexagonal lattices in Caspar–Klug theory.

The cubic protein container designed by Lai et al. (24) (Fig. 1*E*, *Left*) provides a simple example of this interaction network approach. Representing each of its 24 proteins as a node (Fig. 1*E*, *Left*) and drawing connections between interacting proteins, results in the interaction network (shown in black). By aligning the 4-fold and 3-fold symmetry axes of the particle with those of a reference cube (Fig. 1*E*, *Middle*), the interaction network can be mapped onto the cubic surface. The latter is then embedded into a planar tiling by “unfolding” the cubic surface in the plane (Fig. 1*E*, *Right*). Any other particles assembled from the same protein units should exhibit similar local interactions. From a mathematical point of view, this means that their interaction networks can be constructed by working backward from the planar tiling. In particular, any other planar embedding of the cubic surface, obtained via rescaling and reorienting the surface in the plane such that the symmetry axes of the cube again coincide with those of the tiling (*SI Appendix*, Fig. S3), then presents an alternative particle layout. To reconstruct the biological model, vertices have to be replaced by biological units, oriented such that interacting units meet along the edges of the interaction network (Fig. 1*F*). This cage architecture, inferred via our method, has been observed (14, 24), suggesting that our method can indeed be used to predict viable alternative protein container designs.

The ability to predict alternative particle morphologies that can assemble from the same protein unit(s) is important in nanotechnology. It can be used in the context of kinetic models to compute relative ratios of different particle morphologies for different experimental conditions, thus opening up the opportunity to tune experiments to favour production of desired particle types (12). It also informs the reconstruction of less frequent particle types from cryo-EM data in the case of polymorphic assembly and guides the selection of particle morphologies with desired biophysical properties for specific applications (34). In the following, we demonstrate the predictive power of our approach for a more complex system—cages formed from AaLS pentamers—in which nodes in the interaction network represent assembly units composed of multiple protein subunits. This study is motivated by preliminary experimental evidence of cages bigger than the 36-pentamer cage and smaller than the 72-pentamer cage in the assembly of the AaLS pentamers. We therefore used our method to predict other geometrically viable options. However, this analysis illustrates how the interaction network approach can be used to predict and classify particle structures for any system of interest in bionanotechnology.

## Results

### The Building Blocks of the Interaction Network.

**Fig. 2.**
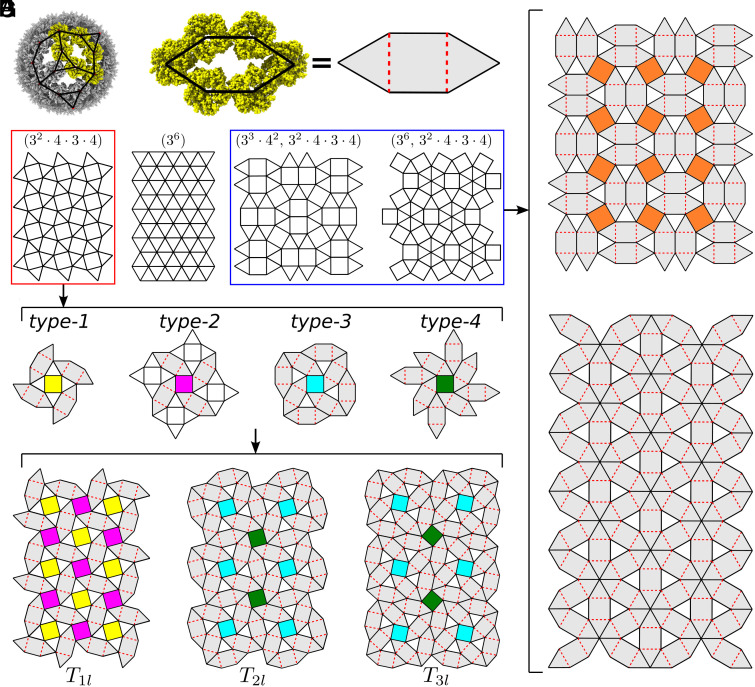
Classification of AaLS protein container architectures based on the interaction network approach. (*A*) The AaLS-13 cage, made of 72 pentamers (PDB: 5MQ7), reveals two types of interactions between pentamers: in groups of three (triangles) and groups of six (squashed hexagons). (*B*) Close-up view of a squashed hexagon and its schematic representation in terms of two triangles and one square; dashed red lines are used to divide the squashed hexagon into a square and two triangles. (*C*) The only k-uniform tilings (up to k=5) that can be partitioned into triangles and squashed hexagons. (*D*) There are four distinct ways in which a 4-fold symmetry axis in the snub square tiling can be surrounded by triangles and squashed hexagons. (*E*) There are only three types of tilings, modulo handedness, that can be constructed from these vertex environments: $T_{1l}$ from type-1 and type-2, and $T_{2l}$ and $T_{3l}$ from type-3 and type-4. (*F*) and (*G*) The unique ways in which the ($3^{3}\cdot 4^{2}$, $3^{2}\cdot 4\cdot 3\cdot 4$) and ($3^{6}$, $3^{2}\cdot 4\cdot 3\cdot 4$) tilings can be partitioned into triangles and squashed hexagons.

Given a nanoparticle of interest, the first step consists in computing its interaction network. In AaLS-based nanoparticles, pentamers and their genetic variants self-assemble into a spectrum of different particles with tetrahedral and icosahedral symmetry (23). Representing the CoMs of the pentamers as nodes and drawing edges between interacting pentamers, we obtain the interaction network (Fig. 2*A*).

The next step in the analysis is to identify the distinct geometric shapes in the interaction network. In this case, there are two types: interactions in groups of three, corresponding to triangles, and interactions in groups of 6, corresponding to nonregular hexagons. The latter, called squashed hexagons in the following because of their characteristic shapes, can be divided into two triangles and one square as indicated by dashed red lines in Fig. 2*B*.

### Planar Tilings Representing the Interaction Network.

The interaction network of AaLS pentamer cages can therefore be embedded into planar tilings made of triangles and squashed hexagons. For other nanoparticles, the nature of tiles will depend on the local interaction patterns and may therefore differ. However, as nanoparticles self-assembling from (potentially multiple different) protein units exhibit only a limited spectrum of distinct local interaction patterns between neighbouring capsomers, planar tilings associated with their interaction networks must all be k-uniform tilings, meaning they are tessellations of the plane with only a limited number (k) of distinct vertex types (cf. *SI Appendix*, Fig. S4 and *Text*). In the case of AaLS-based nanoparticles, we therefore consider k-uniform tilings made of triangles and squares as an intermediate step to obtaining all possible tilings in terms of triangles and squashed hexagons by deleting edges in the triangle-square tilings.

### Classification of Tilings Associated with the Interaction Network.

k-uniform tilings have been classified. There are in total 575 tilings with polygonal faces up to k=5 (*SI Appendix*, *Text*), 140 of which are made entirely of triangles and squares (35–37). The combinatorial task is to identify all possible tilings that are given exclusively in terms of the building blocks of the interaction network, here triangles and squashed hexagons. Note that the tiling can potentially also include a set of squares aligning with the 3-fold symmetry axes of the cage, as these become triangles in the 3D surface (see Fig. 1 *E* and *F* for an example). As tilings in which four squares tessellate a bigger square cannot be subdivided into triangles and squashed hexagons, these tilings are therefore excluded from further analysis, reducing the number of candidates to 58. In order to construct a particle with tetrahedral or octahedral symmetry from such tilings, the symmetry axes of the protein cage must be identified with symmetries in the tiling, as illustrated in Fig. 1 *E* and *F*. This requires the tiling to have 3-fold and/or 4-fold symmetries. Of note, 46 of the 58 tilings have only 2-fold symmetry and therefore cannot be used to construct the surface lattices of particles with tetrahedral or octahedral symmetry. We checked each of the 12 remaining tilings individually, excluding five tilings (*SI Appendix*, Fig. S5*A*) from further consideration (cf. *SI Appendix*, Fig. S6*A* and *Text*). Three of the remaining seven tilings (*SI Appendix*, Fig. S5*B*) contain local 6-fold symmetry axes and therefore must also be excluded as they would require vertices representing pentamers to be positioned on a 6-fold symmetry axis (cf. *SI Appendix*, Fig. S6*B* and *Text*). In summary, only four tilings fulfill all necessary criteria to allow for the embedding of an AaLS interaction network. These are the triangular ($3^{6}$) and the snub square ($3^{2}\cdot 4\cdot 3\cdot 4$) tiling, which are both uniform, and the two 2-uniform tilings ($3^{3}\cdot 4^{2}$, $3^{2}\cdot 4\cdot 3\cdot 4$) and ($3^{6}$, $3^{2}\cdot 4\cdot 3\cdot 4$) (Fig. 2*C*).

For each of these tilings, we next identify all possible ways in which they can be reorganised into triangles and squashed hexagons. First, we focus on the snub square tiling which has 4-fold symmetry axes at the centres of its squares (Fig. 2*C*, *Left*). There are four inequivalent options of organising triangles and squashed hexagons around these axes (Fig. 2*D*). One option is to accommodate four triangles around the square (yellow) and continue with squashed hexagons (type-1). The other option is to organise four squashed hexagons around a square (magenta) and then continue in one of the following ways: either locate triangles between the squashed hexagons (type-2) or accommodate the squashed hexagons in a way to either connect (type-3) or place them between (type-4) the previously added hexagons.

Starting with a type-1 square, the only option is to continue the tiling with type-2 squares (*SI Appendix*, Fig. S7*A*), and vice versa, resulting in the tiling called $T_{1l}$ (Fig. 2*E*) as the unique solution. Starting from a type-3 square, there are two squares where choices have to be made (*SI Appendix*, Fig. S8*A*). In each case, a type-4 square is required next, and then the tessellation must be continued with alternating type-3/type-4 squares (*SI Appendix*, Fig. S8 *B* and *C*), leading to the tilings $T_{2l}$ and $T_{3l}$ (Fig. 2*E*). The same construction can be applied to the right-handed versions of the squares, i.e., the opposite-handed versions obtained using the mirror images of the configurations in Fig. 2*D*. This results in analogous right-handed tilings, denoted as $T_{1r}$ (*SI Appendix*, Fig. S7*B*), $T_{2r}$, and $T_{3r}$ (*SI Appendix*, Fig. S8*D*). Thus, modulo handedness, the snub square tiling can be divided into squashed hexagons and triangles in precisely three inequivalent ways, corresponding to the tilings $T_{\mathrm{jl}}$ (j=1,2,3) shown in Fig. 2*E*.

For all other tilings, the combinatorics are much simpler. The ($3^{3}\cdot 4^{2}$, $3^{2}\cdot 4\cdot 3\cdot 4$) tiling can be divided into triangles and squashed hexagons in only one way (Fig. 2*F*). In the ($3^{6}$, $3^{2}\cdot 4\cdot 3\cdot 4$) tiling, placement of the 6-fold vertices must be such that the particle generated from the tiling does not contain any 6-fold symmetric vertices as this would be incompatible with vertices representing pentamers. Thus, this tiling can only be subdivided into triangles and squashed hexagons in a unique way (Fig. 2*G*).

### Construction of Protein Cage Architectures.

**Fig. 3.**
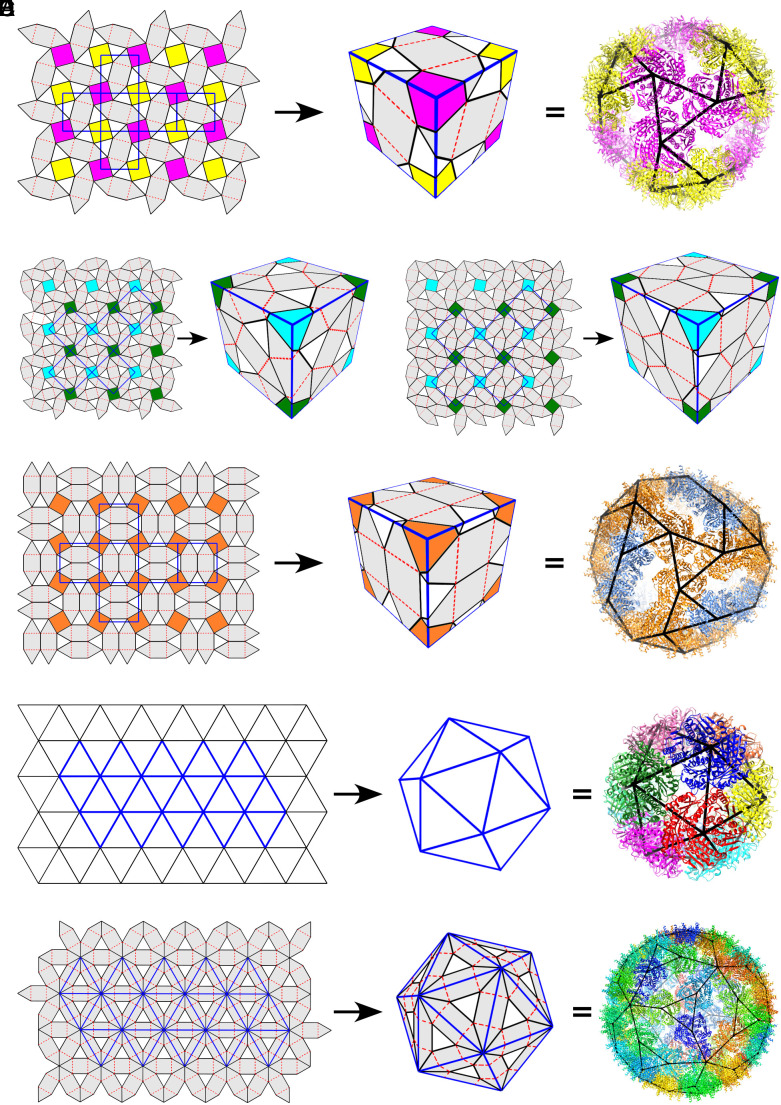
AaLS cage architectures derived from interaction networks. (*A*) The $T_{1l}$ tiling provides the layout of a particle made from 24 pentamers and corresponds to the surface structure of a known AaLS protein cage (PDB: 7A4F). (*B*) and (*C*) Particle architectures derived from the $T_{2l}$ and $T_{3l}$ tilings correspond to cages with tetrahedral symmetry made from 48 and 60 pentamers, respectively. These cages have not been reported to date. (*D*) The ($3^{3}\cdot 4^{2}$, $3^{2}\cdot 4\cdot 3\cdot 4$) tiling predicts a particle made from 36 pentamers with tetrahedral symmetry. Its surface architecture corresponds to a known AaLS protein cage (PDB: 5MQ3). (*E*) The triangular tiling corresponds to a polyhedron with 12 vertices, which embodies the architecture of the WT AaLS cage (PDB: 5MPP). (*F*) The ($3^{6}$, $3^{2}\cdot 4\cdot 3\cdot 4$) tiling corresponds to an icosahedral particle made from 72 pentamers and corresponds to the AaLS-13 protein cage (PDB: 5MQ7).

Given the exhaustive list of tilings embodying the characteristics of the interaction network derived above, models for particles with tetrahedral or octahedral symmetry can then be constructed via different embeddings of a cubic surface (*SI Appendix*, Fig. S3). For this, a planar representation of the cubic surface must be embedded into the tiling such that its corners align with the centres of appropriately spaced squares in the tiling. For example, allocating the vertices of the cubic surface to the centres of adjacent coloured squares in the $T_{1l}$ tiling (Fig. 3*A*, *Left*), and then mapping these onto the vertices of a cube (Fig. 3*A*, *Middle*), generates a model for an AaLS cage made of 24 pentamers (Fig. 3*A*, *Right*). This particle has been observed in the self-assembly of AaLS pentamers (38).

It is not possible to embed the surface of a cube into this tiling in any other way without mapping squares in the tiling onto the faces of the cube (cf. *SI Appendix*, Fig. S9*A*). As vertices represent pentamers, this would generate a ring-like interaction between four AaLS pentamers in the particle surface, which is a local interaction type that has not been observed in any AaLS cage to date. We therefore exclude it from our classification. There are thus no other biologically viable AaLS cage models that can be constructed from this tiling. Similarly, both $T_{2l}$ and $T_{3l}$ result in only one tetrahedral model each. The former corresponds to a protein cage made from 48 pentamers (Fig. 3*B*), and the latter to a 60-pentamer cage (Fig. 3*C*). These particles have not yet been observed but are consistent with the experimentally observed local interaction rules and therefore provide viable geometric models for AaLS cages. It is possible that these cages have previously been overlooked because they occur less frequently during polymorphic assembly than other variants. Note that all known AaLS cages only exhibit the left-handed version of the tilings. We therefore only consider the left-handed versions in our analysis, assuming that the interactions in all cages should have similar characteristics.

The 2-uniform tiling ($3^{3}\cdot 4^{2}$, $3^{2}\cdot 4\cdot 3\cdot 4$) has only one type of 4-fold symmetry axis, shown in orange (Fig. 3*D*). In analogy to the snub square tiling, only the smallest embedding of the cubic surface is possible and is obtained by associating neighbouring squares with the vertices of a cubic face (Fig. 3*D*). This particle is made from 36 pentamers that are organised with tetrahedral symmetry and corresponds to one of the AaLS pentamer cages reported previously (23). Note that, as before, any larger models, obtained via an embedding of a rescaled cubic surface (*SI Appendix*, Fig. S9) would necessarily contain a square, i.e., a group of four pentamers, and we again reason that this would not be a biologically viable option.

The remaining two tilings include 6-fold symmetry axes. It is therefore possible to construct particle architectures with icosahedral symmetry from them following the Caspar–Klug construction (19). The smallest particle with icosahedral symmetry that can be derived from the triangular tiling is the icosahedron, a polyhedron with 12 vertices. This model corresponds to the wild-type (WT) AaLS cage (Fig. 3*E*). In the Caspar–Klug construction, higher-order triangulations are possible in which the faces of the icosahedron are subdivided into triangular facets. However, in their surface lattice interpretation, proteins are allocated to the $60^{{}^{\circ}}$ angles of the triangular facets, thus generating models with 12 pentamers and otherwise hexamers. Here, on the other hand, the triangulation is representing the interaction network between pentamers, whose positions are indicated by the vertices. Therefore, larger particles with icosahedral symmetry are not feasible as they would map pentamers onto 6-coordinated vertices in the triangulation. Similarly, tetrahedral and octahedral particles can be constructed via triangular surface lattices (26) but are not viable in the framework of AaLS interaction networks as they would locate pentamers on 3- or 4-fold symmetry axes. Similar arguments show that there exists only one planar embedding of an icosahedral surface into the ($3^{6}$, $3^{2}\cdot 4\cdot 3\cdot 4$) tiling leading to a viable AaLS cage (Fig. 3*F*). The resulting cage morphology, a particle made from 72 pentamers, corresponds to the AaLS-13 cage (23).

There are thus precisely six cage structures with 3D symmetry that can self-assemble according to the known local interaction pattern of the AaLS pentamers (Fig. 3 and Table 1), ranging in size from the WT AaLS cage (12 pentamers) to the AaLS-13 cage (72 pentamers). Our classification implies that there are no AaLS particles with octahedral symmetry. It also predicts intermediate structures formed from 48 and 60 pentamers that have not been reported to date. Note that the distinct symmetry types observed here for AaLS cage assembly can also occur in the self-assembly of other protein cages (39, 40). Our analysis demonstrates how tilings encoding the interaction network of a protein cage can be used to systematically enumerate all possible alternative protein cage structures with 3D symmetry that can also assemble from the same protein units. While we have demonstrated our approach for the AaLS system, it can readily be applied to any protein nanocontainer architecture of interest following the methodology introduced here. This analysis is thus a primer for the modelling of protein nanocontainers in bionanotechnology.

**Table 1.** Classification of AaLS protein cages with cubic symmetry (9)

Tiling	No. of pentamers	Symmetry	Experimental observation	Figure
Triangular ($3^{6}$)	12	Icosahedral	Observed (PDB: 5MPP from ref. 23)	Fig. 3*E*
Snub square tiling ($3^{2}\cdot 4\cdot 3\cdot 4$) $T_{1l}$	24	Tetrahedral	Observed (PDB: 7A4F from ref. 38)	Fig. 3*A*
($3^{3}\cdot 4^{2},3^{2}\cdot 4\cdot 3\cdot 4$)	36	Tetrahedral	Observed (PDB: 5MQ3 from ref. 23)	Fig. 3*D*
Snub square tiling ($3^{2}\cdot 4\cdot 3\cdot 4$) $T_{2l}$	48	Tetrahedral	Not observed	Fig. 3*B*
Snub square tiling ($3^{2}\cdot 4\cdot 3\cdot 4$) $T_{3l}$	60	Tetrahedral	Not observed	Fig. 3*C*
($3^{6},3^{2}\cdot 4\cdot 3\cdot 4$)	72	Icosahedral	Observed (PDB: 5MQ7 from ref. 23)	Fig. 3*F*

## Discussion

Protein containers, either adapted from naturally occurring protein cages or de novo designed, are pillars of bionanotechnology. Many groups worldwide are developing novel types of nanoparticles for a host of applications, for example, using the Rosetta Software (6, 41–43). The simultaneous assembly of a wide spectrum of particle morphologies—a phenomenon known as particle polymorphism—poses a challenge for nanocontainer production. Such polymorphism, which has also been observed in the assembly of capsid proteins in the presence and absence of viral RNA genomes (44, 45), is often triggered by genetic modifications of the capsid protein subunit. This includes insertion of amino acid sequences (SpyTags) into the outward-facing portion of the protein subunits, a method that is standardly used to functionalise the particle surface (using SpyCatchers) with antigens for vaccine production. Such modifications are known to result in the assembly of multiple different particle morphologies that contain the WT morphology as only one of many distinct options (12). Genetic modifications to alter the chemical properties of the protein units, such as their charges or sensitivity to pH, have similar effects, both in nanocontainers derived from bacterial enzymes (23, 46–48) and in virus-like particles (44, 49). Understanding the determinants of this particle polymorphism is an important step in controlling the assembly outcome.

We introduce here a theoretical framework to characterise the spectrum of nanoparticle morphologies that can assemble from a given set of protein units. This interaction network approach can be used widely in protein nanotechnology, extending previous approaches (19, 26, 29, 50–54) and related models that characterise the surface architectures of de novo designed nanoparticles used as malaria vaccines (55). These approaches fail because the existence of protein subunits not interacting with neighbouring capsomers makes it difficult to define a biologically meaningful mathematical unit for the tiling models. The method introduced here closes this gap in our understanding of protein nanocontainer architecture. It uses knowledge of the local interactions between the self-assembling capsomers to systematically enumerate all viable container designs with 3D symmetry that can be formed from them. The predictive power of this approach is demonstrated for particles formed from AaLS pentamers (23, 38), for which our method not only characterises all experimentally observed variants of different sizes and 3D symmetries, but also predicts structures that have not been observed yet. As tiles in CK theory and VTT represent capsomers, the dual tilings—obtained by replacing tiles by vertices and connecting vertices corresponding to adjacent tiles—can also be viewed as interaction networks (*SI Appendix*, Fig. S10). Thus, the interaction network approach provides a unifying framework for the modelling of virus and nanoparticle architecture alike. It is applicable in bionanotechnology for the classification of nanocontainer architecture and can be built as local constraints into programmes like Rosetta to support nanoparticle design. The geometric models also open up broad avenues for the study of the biophysical properties of protein cages, such as their propensity for fragmentation and cargo release (34), or their kinetics of self-assembly along assembly pathways leading to distinct particles (12). Such models predict the relative ratios of different particle types depending on experimental conditions, revealing how assembly can be biased towards specific outcomes. This, in turn, provides a means of increasing the yield of desired particles, supporting the rational design of protein containers for diverse applications in bionanotechnology.

## Supplementary Material

Appendix 01 (PDF)

## Data Availability

All study data are included in the article and/or *SI Appendix*.
