# Supplementary material for: An interaction network approach predicts protein cage architectures in bionanotechnology
Source: Proc Natl Acad Sci U S A. 2023 Dec 7;120(50):e2303580120. doi: 10.1073/pnas.2303580120 (PMC10723117; doi:10.1073/pnas.2303580120)
Supplement: Supplementary file 1 — Appendix 01 (PDF) [file pnas.2303580120.sapp.pdf]

Supporting Information for

An interaction network approach predicts protein cage architectures in bionanotechnology

Farzad Fatehi and Reidun Twarock

Corresponding authors: Farzad Fatehi and Reidun Twarock
E-mails: ffc505@york.ac.uk and rt507@york.ac.uk

This PDF file includes:

Supporting text
Figs. S1 to S10
SI References

Supporting Information Text

Tiling models of protein container architecture - definitions and concepts

The first tiling models of viral protein containers were introduced by Caspar and Klug. They used hexagonal lattices and triangulations to pinpoint the positions of capsid proteins in quasi-equivalent positions, i.e. occupy similar local environments in the capsid shell. These lattices are special cases of uniform tilings: A **uniform tiling** is a tessellation of the plane by convex regular polygons that is vertex-transitive, i.e., all of its vertices are equivalent under the symmetries of the tiling.

There are 11 uniform tilings, also called Archimedean lattices. As all vertices of a uniform tiling are surrounded by identical arrangements of polygons, this feature is used to label different options. For example, the Kagome lattice (Fig. S4A) is a uniform tiling in which each vertex is surrounded in clockwise or anti-clockwise order by a triangle, hexagon, triangle and hexagon. It is therefore denoted as $(3 \cdot 6 \cdot 3 \cdot 6)$, where numbers refer to the numbers of corners in each polygon in successive order.

In the context of the interaction network approach, we also allow for capsid architectures that violate the quasi-equivalence principle. In this case, there can be more than one type of vertex environment, and we therefore need k-uniform tilings: A **k-uniform tiling** of the plane is a tessellation by convex regular polygons, connected edge-to-edge, with k distinct types of vertex environments. In analogy to uniform tilings, they are labelled by k indices characterising their distinct vertex environments. For example, the vertices of the 2-uniform tiling in Figure S4B are either surrounded by three triangles and two squares (type $(3^3 \cdot 4^2)$, black disk), or two triangles, a square, a triangle and a square (type $(3^2 \cdot 4 \cdot 3 \cdot 4)$, grey disk), and this tiling is therefore labelled as $(3^3 \cdot 4^2, 3^2 \cdot 4 \cdot 3 \cdot 4)$. k-uniform tilings have been enumerated, and there are 20 2-uniform tilings, 61 3-uniform tilings, 151 4-uniform tilings and 332 5-uniform tilings, thus 575 in total up to $k = 5$.

Arguments for the exclusion of tilings from the classification

The classification of AaLS surface lattices relies on the identification of k-uniform tilings that can be partitioned into triangles and squashed hexagons. Starting with all 140 tilings given in terms of triangles and squares, we derive tilings in terms of triangles and squashed hexagons (the characteristic local interactions of AaLS cages) by deleting edges. Here we elaborate on two arguments that are used to exclude specific types of k-uniform tilings (cf. main text p.4):

Exclusion of tilings that cannot be partitioned into triangles and squashed hexagons: Figure S5A shows tilings that cannot be partitioned exclusively into triangles and squashed hexagons and are therefore excluded from our analysis. Figure S6A illustrates why this is the case based on the example of the 3-uniform tiling $(3^6, 3^3 \cdot 4^2, 3^2 \cdot 4 \cdot 3 \cdot 4)$. The square marked by a star cannot be the centre of a 4-fold symmetry axis, and a squashed hexagon can be placed on it in only one way as shown in grey. Now the square marked by a disk cannot be the centre of a 4-fold symmetry axis, and no squashed hexagon can be placed on it, thus this tiling cannot be converted into a tessellation by triangles and squashed hexagons via deletion of edges. An similar argument excludes all five tilings in Fig.S5A.

Exclusion of tilings containing local 6-fold symmetry axes: Figure S5B shows tilings that can be divided into triangles and squashed hexagons, but contain local 6-fold symmetry axes. As vertices in the tilings represent pentamers, and pentamers cannot be placed on a 6-fold symmetry axis, these tilings are also excluded. Fig. S6B illustrates this for the 5-uniform tiling $(3^6, (3^2 \cdot 4 \cdot 3 \cdot 4)4)$: Even though it is possible to identify 4-fold symmetry axes (Fig. S5B, yellow squares) that could align with those of a cube, even the smallest square face would necessarily contain a local 6-fold symmetry axis (red dot), which is not compatible with the interpretation of vertices as locations of pentamers. Similar arguments also apply to the other two tilings in Fig. S5B.

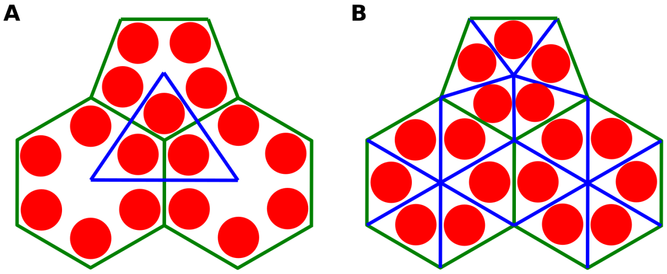

Fig. S1. Distinct tiling types predict different orientations of the capsomers (assembly units, usually composed of several protein subunits) in the capsid surface. A triangulation modelling Pariacoto virus (A), and a rhomb tiling representing bacteriophage MS2 (B), predict different relative positions of the protein subunits (red dots). Locating protein positions in the corners of the triangular or rhomb tiles (blue) following the convention in Caspar-Klug theory, the resulting capsid blueprints have different orientations with respect to the underlying pentagonal/hexagonal lattice architecture. In the triangulation pairs of protein subunits, and in the rhomb tiling individual protein subunits, in neighbouring hexamers are facing each other.

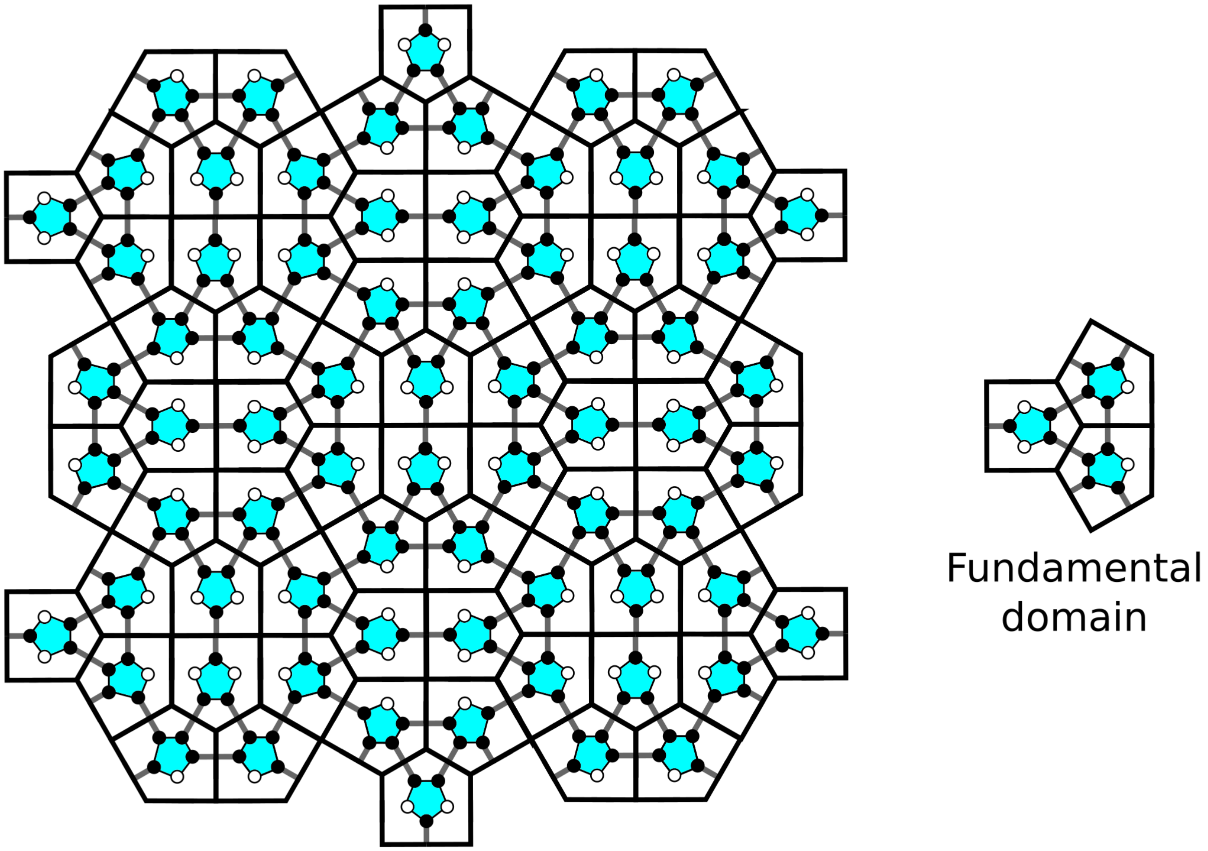

Fig. S2. Surface tessellation of the 36-pentamer AaLS cage architecture in terms of Voronoi cells. The fundamental domain (or asymmetric unit) of the tiling consists of three Voronoi cells (right). Pentamers are coloured in cyan, and black and white circles indicate monomers that bind, and respectively do not bind, to a protein subunits of an adjacent pentamer.

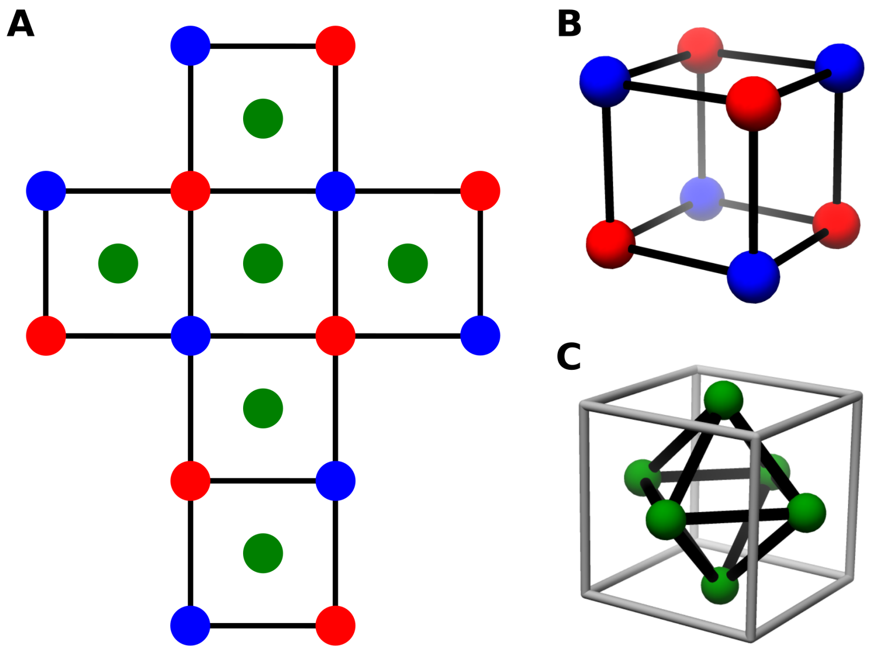

Fig. S3. Deriving tetrahedral and octahedral symmetries from a cubic net. (A) A planar embedding of the surface of a cube. If all vertices are identical, the cube has octahedral symmetry, but colouring its vertices in red and blue to match the cube in (B) reduces it to tetrahedral symmetry. (C) As the octahedron is the dual of the cube, both have the same symmetry. The vertices of the octahedron (green) correspond to the 4-fold symmetry axes of the cube.

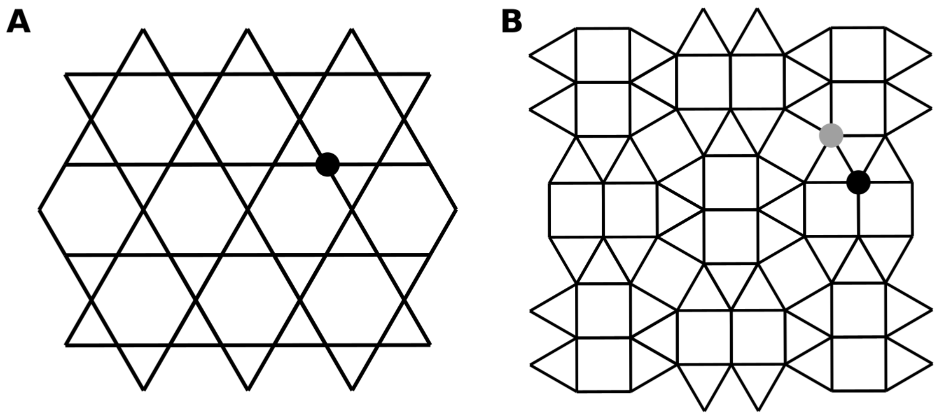

Fig. S4. Examples of a uniform and a k-uniform tiling. (A) A uniform tiling is vertex-transitive, i.e. all its vertex environments are identical, as indicated here by a black disk in the $(3 \cdot 6 \cdot 3 \cdot 6)$ tiling. (B) In a 2-uniform tiling, each vertex adopts of one of only two distinct types. In the $(3^3 \cdot 4^2, 3^2 \cdot 4 \cdot 3 \cdot 4)$ tiling shown, $(3^3 \cdot 4^2)$ is indicated by a black disk, and $(3^2 \cdot 4 \cdot 3 \cdot 4)$ by a grey disk.

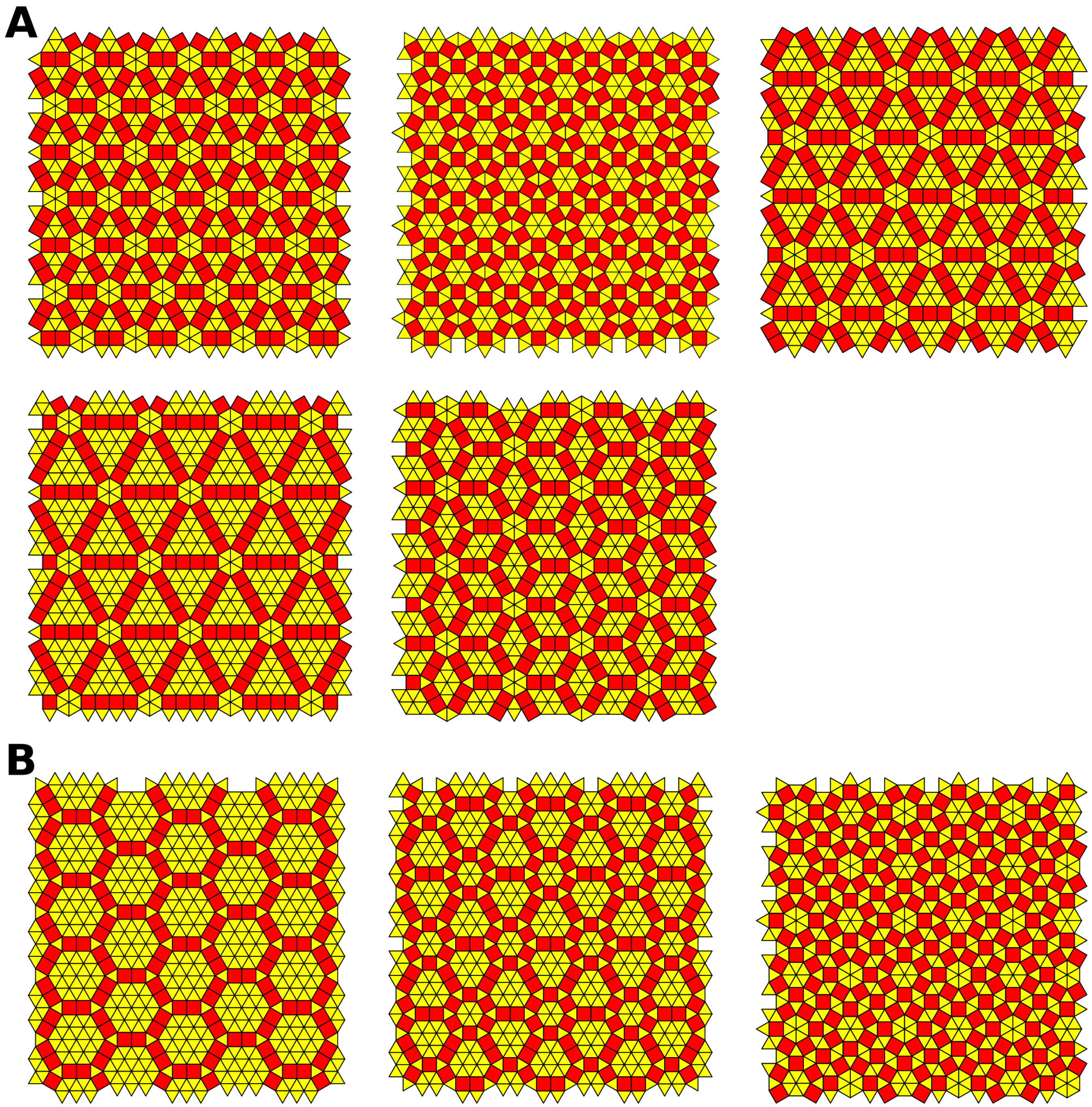

Fig. S5. Examples of uniform tilings that do not correspond to surface lattices of AaLS cages. (A) Tilings that cannot be reorganised into triangles and squashed hexagons do not reflect the local interaction pattern; and (B) tilings that generate spherical particles containing a local 6-fold symmetry axis are incompatible with vertices representing pentamers. Tiling figures are adapted from work by Tom Ruen (https://commons.wikimedia.org/wiki/User:Tomruen) (1).

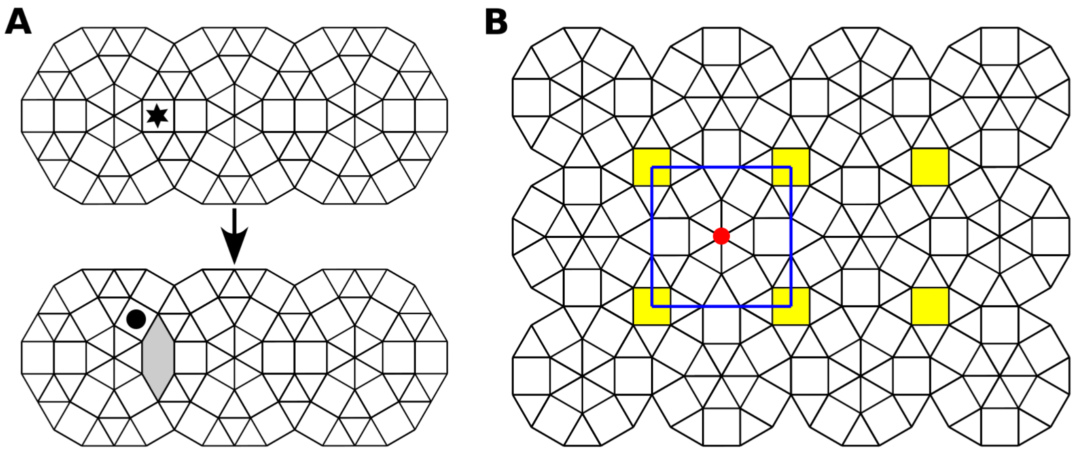

Fig. S6. Graphical illustrations of the arguments used to exclude specific tiling types from the classification of AaLS cage architectures. (A) Partitioning the 3-uniform tiling (3^6, $3^3 \cdot 4^2$, $3^2 \cdot 4 \cdot 3 \cdot 4$) into triangles and squashed hexagons is not possible as the centre of the square marked by a disk cannot be the location of a 4-fold symmetry axis, nor can a squashed hexagon be placed on it. (D) The smallest cubic surface that can be embedded into the 5-uniform tiling (3^6, $(3^2 \cdot 4 \cdot 3 \cdot 4)$) contains a vertex on a local 6-fold symmetry axis (red dot). As vertices indicate pentamer positions, it is not possible to construct an AaLS cage from this tiling.

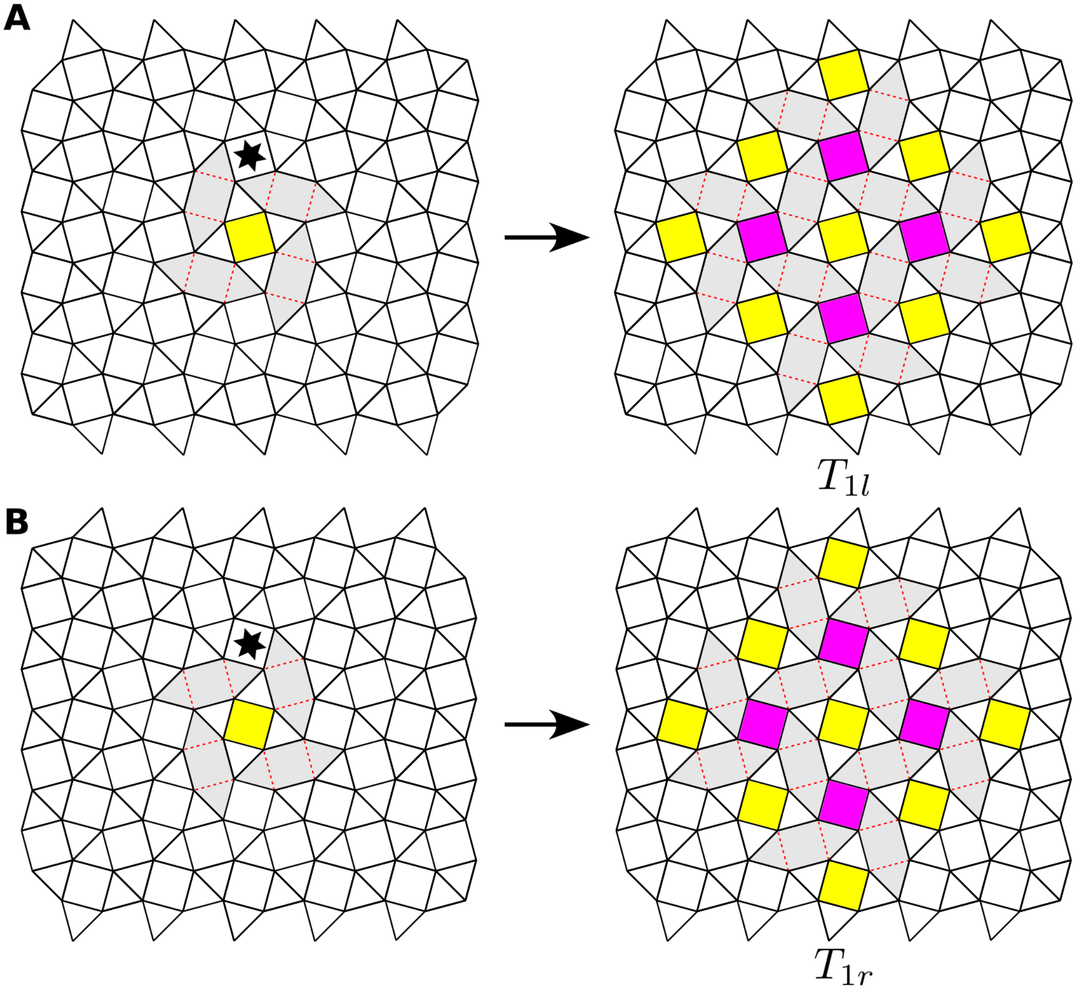

Fig. S7. AaLS surface models of opposite handedness. For each tiling in our classification there is a right-handed counterpart that is its mirror image in the plane. For example, the T_{1l} and T_{1r} tilings implied by the arrangements of triangles and squashed hexagons around a left-handed (A) and right-handed (B) *type-1* symmetry axis are mirror images of each other. The squares marked by stars must correspond to left-handed (A) or right-handed (B) *type-2* squares.

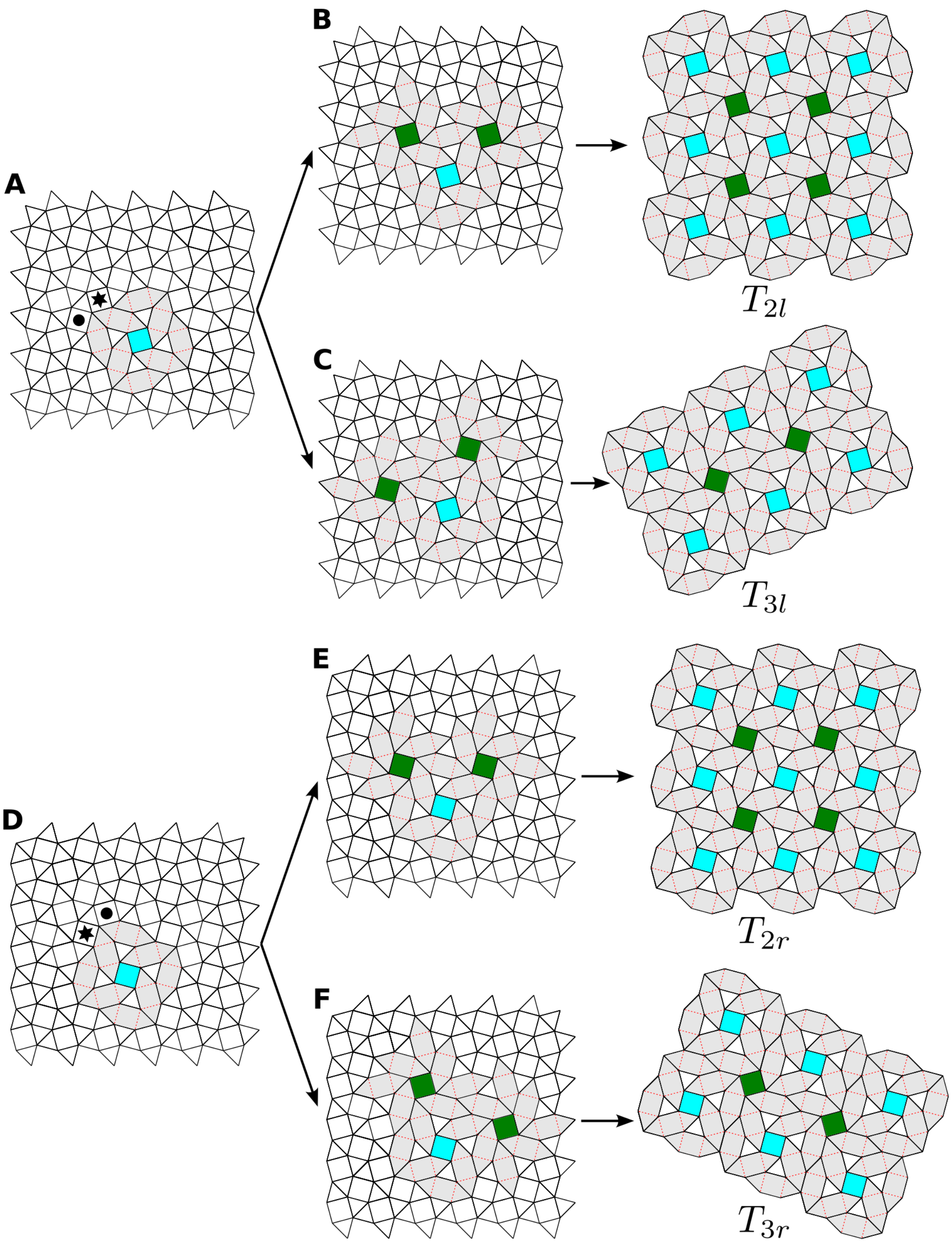

Fig. S8. Derivation of AaLS surface models corresponding to *type-3* and *type-4* squares of opposite handedness. Starting from a left-handed (A) and right-handed (D) *type-3* 4-fold symmetry axis, either the square indicated by a disc or by a star must be a *type-4* square, resulting in the left-handed (B and C) and right-handed (E and F) options, respectively. Each of these can only be continued in a unique way, resulting in left-handed tilings T_{2l} and T_{3l}, and right-handed counterparts T_{2r} and T_{3r}.

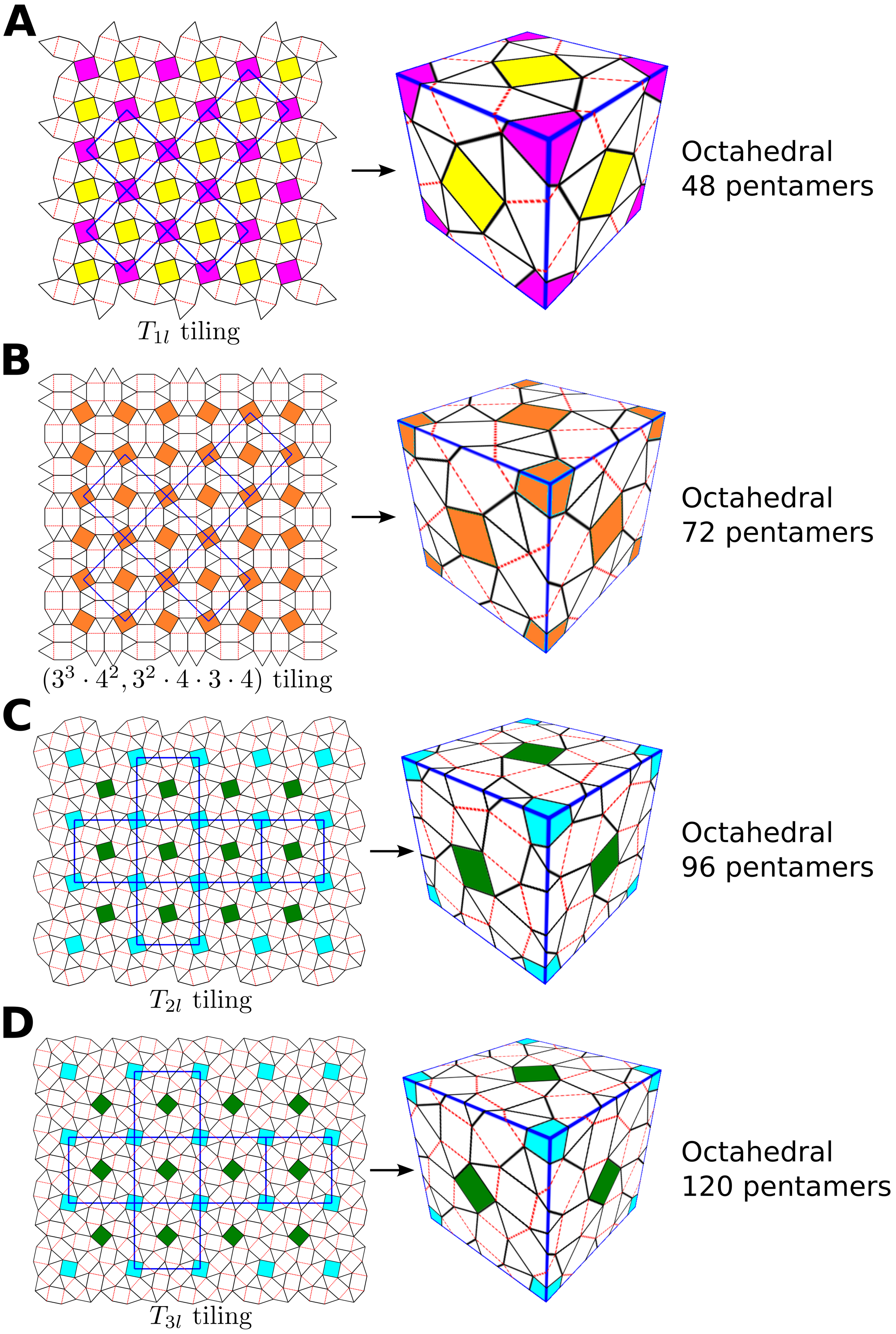

Fig. S9. Construction of the second smallest particle layouts from the tilings with 4-fold symmetry axes. Embeddings of rescaled versions of the cubic surface result in octahedral particles that each contain a square in their faces (yellow (A), orange (B), and green (C and D)). As vertices represent pentamers, this would correspond to a hole surrounded by four pentamers interacting with each other in a circular arrangement, which is a local interaction pattern that has not been observed experimentally. Thus, we reason that these particles are not biologically viable options. However, they might be engineered if pentamers are mutated to enable this type of local interaction.

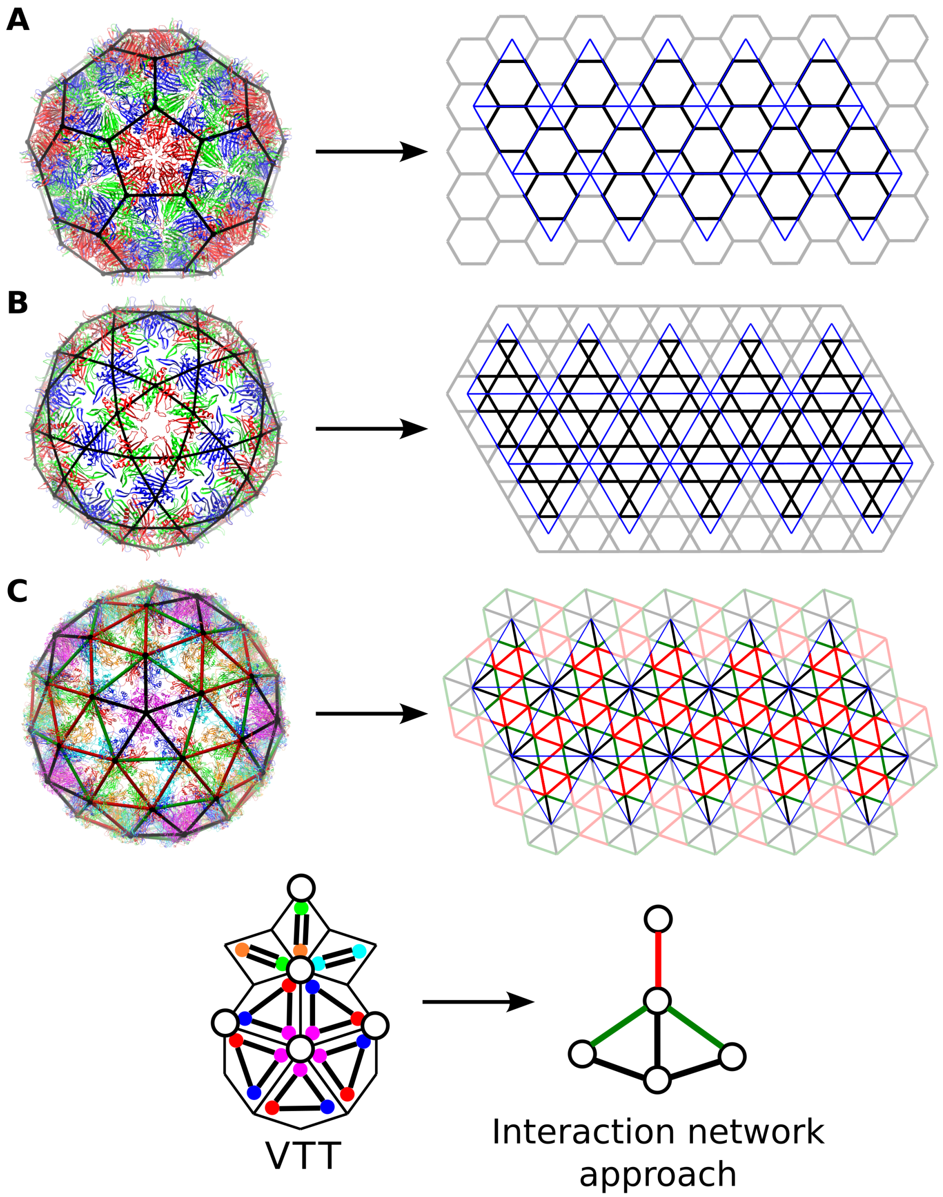

Fig. S10. The tiling models in Caspar-Klug and Viral Tiling theory can also be represented by interaction networks. (A) A hexagonal lattice, connecting the midpoints of the triangular tiles representing the Pariacoto virus capsid (Fig. 1A; PDB: 1F8V), is an example of an interaction network associated with a Caspar-Klug model. (B) The Kagome lattice, consisting of hexagonal and triangular faces, is the interaction network of Bacteriophage MS2 (PDB: 2MS2), which is represented by a rhomb tiling in Viral Tiling theory (VTT). (C) The Human Papilloma virus capsid (PDB: 3J6R), in which protein subunits interact via dimer and trimer interactions, is modelled in terms of rhomb and kite tiles in VTT. Its interaction network is a weighted triangular tiling, in which different types of interactions between pentamers are shown colour-coded. In particular, as the close up at the bottom shows, the dimer and trimer interactions between protein subunits give rise to three distinct interactions between pentamers in the interaction network: red edges correspond to dimer interactions, i.e. interactions represented by a rhomb tile in VTT; green edges indicate interactions within a trimer, i.e. one kite tile; and black edges represent interactions in two adjacent trimers, i.e. two neighbouring kite tiles.

References

1. Tiling figures created by Tom Ruen - Own work, CC BY-SA 4.0, 2015, Links:
https://commons.wikimedia.org/w/index.php?curid=40895573,
https://commons.wikimedia.org/w/index.php?curid=40895575,
https://commons.wikimedia.org/w/index.php?curid=41002117,
https://commons.wikimedia.org/w/index.php?curid=41002126,
https://commons.wikimedia.org/w/index.php?curid=41414681,
https://commons.wikimedia.org/w/index.php?curid=41414688,
https://commons.wikimedia.org/w/index.php?curid=41414692,
https://commons.wikimedia.org/w/index.php?curid=41414694.
